# Anti-Proliferative Effect of Doxorubicin-Loaded AS1411 Aptamer on Colorectal Cancer Cell

**DOI:** 10.31557/APJCP.2021.22.7.2209

**Published:** 2021-07

**Authors:** Walaiporn Lohlamoh, Boonchoy Soontornworajit, Pichayanoot Rotkrua

**Affiliations:** 1 *Division of Biochemistry, Department of Preclinical Science, Faculty of Medicine, Thammasat University, Pathumthani, 12120, Thailand. *; 2 *Department of Chemistry, Faculty of Science and Technology, Thammasat University, Pathumthani, 12120, Thailand. *

**Keywords:** AS1411 aptamer, colorectal cancer, doxorubicin, nucleolin

## Abstract

**Background:**

Doxorubicin (Dox) inhibits DNA replication and causes DNA damage resulting in cell death. It is a common drug for treatment of many cancers. Treatment efficacy and side effects of Dox are critical issues in using it because the drug lacks of specificity. The objective of this study was to improve the specificity of Dox by the incorporation of this drug with AS1411 aptamer (ASA).

**Methods:**

Dox was intercalated into the duplex sites of ASA, a recognition molecule for a number of cancer cells, and formed Dox-loaded ASA. The recognition ability proceeded through specific binding between the aptamer and nucleolin overexpressed in the cancer cells. The tested cells were human colorectal adenocarcinoma cell line (SW480) and human normal colon cell CCD841 CoN (CCD841). Binding of ASA to the cells was tested using flow cytometer and fluorescence microscope. Intercalation of Dox into DNA duplex was confirmed by fluorescence spectrometry. Effect of ASA, Dox, and Dox-loaded ASA on cell viability was examined by cell proliferation assay. Caspase-3 activation was analyzed by western blotting.

**Results:**

ASA bound specifically to SW480 cells via interaction between the aptamer and nucleolin because the nucleolin was highly expressed in SW480 cells. ASA decreased the viability of SW480 cells in a dose-dependent manner. Dox was more toxic than ASA. Fluorescence quenching revealed that Dox was able to intercalate in base pairing sites of the aptamer. Dox-loaded ASA inhibited the proliferation of SW480 cells, because the aptamer facilitated the Dox uptake into these cells which caused the cell apoptosis, indicated by the significant decrease in procaspase-3, apoptosis marker protein.

**Conclusion:**

This study succeeded to prepare Dox-loaded ASA by intercalation of the drug that inherited the binding function from the aptamer and anti-cancer activity from Dox. Dox-loaded ASA showed promise for effective cancer treatment with lower level of side effects.

## Introduction

Doxorubicin (Dox), commercially named as Adriamycin, was extracted from *Streptomyces peucetius *and was first used in clinical trials in the 1960s. It is a compound that has anthracycline group with a four-member ring system containing a chromophore, anthraquinone, and aminoglycoside (Cutts et al., 2005). Dox is a common chemotherapeutic drug for treating a number of cancers including bladder, ovarian, and lung cancer (Al-malky et al., 2020). Anticancer action of Dox is achieved by the intercalation of this drug in the genetic materials. The intercalation undergoes mainly at GC-rich sites, which causes the malfunction of topoisomerase-II, an enzyme which relaxes supercoils in DNA for transcription. After forming Dox-topoisomerase-II complex, the process of DNA replication is inhibited (Pommier et al., 2010). Another mechanism is that Dox induces oxidative stress resulting in reactive oxygen species (ROS) in cells (Yurtcu et al., 2015), and increases the exposure of naked DNA resulting in DNA damage and cell death (Tewey et al., 1984). Side effects of Dox are nausea, vomiting, cardiotoxicity, immune suppression, unusual tiredness, weakness, and red coloration of urine (Wadler and Schwartz, 1990). To improve therapeutic efficiency and overcome those side effects, a number of strategies have been proposed and investigated. For instance, a combination between Dox and curcumin enhanced the anti-cancer activity in colorectal cancer (CRC) cells (Khameneh et al., 2019). In the issue of cardiotoxicity, Dox was combined with Dexrazoxane, a cardioprotective agent, and they were administered to patients (Wakharde et al., 2018). A PEGylated liposomal Dox formulation showed promising results in reducing the toxicity and maintaining anticancer activity of Dox (Franco et al., 2018). The ability of Dox to differentiate between cancer and normal cells was another major concern in using this drug effectively. Many recognition molecules such as small biomolecules, peptides, and antibodies have been used to improve the specificity of Dox to its target sites. For instance, folic acid, a small molecule, was incorporated to a magnetic nano-carrier which exhibited the selective delivery of Dox to (CRC) (Martin et al., 2021). Dox-encapsulated nanomicelles that were decorated with a peptide ligand targeting CD36 receptor showed promise of recognizing CD36-overexpressing cell lines (Zheng et al., 2021). The antibody specific to epidermal growth factor receptor helped nanoparticles to deliver Dox to the breast cancer cells in both *in vitro* and *in vivo* tests (Dorjsuren et al., 2020). Although these recognition molecules showed encouraging results, there were major concerns needing to be addressed. Because the affinity of the small molecules and peptides to their targets is within nano to micro molar range, a large quantity of these molecules were required to be incorporated into the delivery system (Hoppe-Seyler et al., 2004). This could limit the potential fabrication of the systems. The application of antibodies was highly limited by their fragile structure (Wu and Senter, 2005), and immunogenicity (Presta, 2006). Therefore, the search for the molecules that have high specificity and affinity and can be used for integrating with cancer therapeutic drugs has drawn much attention to this field. 

Aptamers are short single-stranded oligonucleotides (e.g. DNA or RNA) with length between 20-200 nucleotides. The aptamers are generally developed from SELEX (Song et al., 2012). They form unique three-dimensional structures such as single-stranded segment, internal loop, triplex, G-quadruplex, hairpin and bulge (Sullivan et al., 2019) which provide proper sites for binding to a number of targets including nucleic acids, proteins, viruses, bacteria, whole cells, and small molecules (Mann et al., 2005; Li et al., 2014; Rong et al., 2016; Alizadeh et al., 2017; Carvalho et al., 2019; Wang et al., 2019; Zou et al., 2019). The key binding interactions are van der Waals forces, hydrogen bonding, and electrostatic interaction. Moreover, the aptamers rarely induce immune response. They have been applied in a number of applications including antivirus (Ghobadloo et al., 2014) anticancer (Ireson and Kelland, 2006) and drug delivery (Zhang et al., 2011). Previously, our study has demonstrated that using PDGF-BB aptamer to interfere with the binding of PDGF-BB to its receptor suppressed CRC cell proliferation in part via down-regulation of the Ras/Raf/MEK/ERK signaling pathway (Sae-Lim et al., 2019). AS1411 aptamer (ASA) is the most common aptamer that has been investigated in cancer treatment because it binds to nucleolin overexpressed on plasma membranes of many cancer cells (Bates et al., 1999). It is a single-stranded DNA and a guanosine-rich molecule with a length of 26 mers that forms a variety of G-quadruplex structures (Fan et al., 2016). As a therapeutic agent, ASA has anti-proliferative effect on several cancer cells (Reyes-Reyes et al., 2010; Rosenberg et al., 2014). Moreover, it was developed for delivering drugs specifically to tumors by conjugating this aptamer with several materials such as gold nanoparticles (Kabirian-Dehkordi et al., 2019), liposomes (Liao et al., 2015), and polymeric micelles (Li et al., 2017). These modified materials were able to bind specifically to cancer cells and tumor tissues (Reyes-Reyes et al., 2015). For example, Malik (2015) revealed that ASA-linked gold nanospheres (ASA-GNS) were more effective in cellular uptake and they could enhance the anti-proliferative/cytotoxic effects compared to an unmodified ASA sequence. In addition ASA-GNS inhibited cancer growth but showed no toxicity to the normal cells. However, a direct usage of Dox and ASA for CRC treatment has not been well documented. 

CRC is the third most common cancer found in men and women and it has high incidence and mortality rate (Bray et al., 2018). The development of CRC initiates as a growth of tissues called a polyp inside colon or rectum, a part of the digestive system. Subsequently, the cells divide abnormally, and they are able to invade other tissues. Cancer cells are able to spread to other parts of the body through the blood and lymph system (Howlader et al., 2021). CRC has several potential risk factors such as genetics, environment, lifestyle (e.g. lack of physical exercise, addiction of alcoholic beverage, and smoking), dietary behavior (e.g. consuming high amounts of fat, red meat, and processed meats), and other diseases such as Crohn’s disease and ulcerative colitis. Many researchers have aimed to develop novel strategies, and efficient drug delivery for treating CRC. This also inspires us to study a chemotherapeutic formulation based on ASA and Dox (Dox-loaded ASA) for CRC treatment. This study proposed that ASA provided the intercalation sites for Dox and brought the drug to a target of CRC cells. The CRC and normal control cells were human colorectal adenocarcinoma cell line (SW480) and human colon cell CCD 841 CoN (CCD841), respectively. Binding of ASA to CRC cells was investigated by flow cytometry and fluorescence microscopy. Cytotoxicity of Dox, ASA, and Dox-ASA complex was determined by cell viability test. Apoptotic pathway was investigated by western blot analysis.

## Materials and Methods

*Reagent*

AS1411 aptamer (ASA) and non-binding oligonucleotide (NBO) were purchased from Integrated DNA Technologies (USA), and their sequences were tabulated in Table 1. They were dissolved in sterile distilled water at a concentration of 100 µM and stored at -20°C. Doxorubicin hydrochloride (Dox) was purchased from Fresenius Kabi, Thailand. The following rabbit polyclonal antibodies were purchased from Cell Signaling Technology (USA): caspase-3 (#9662), and beta actin (#4967). These antibodies were prepared at 1: 1,000 dilution in the diluent solution provided by the manufacturer. Odyssey blocking buffer and goat anti-rabbit IRDye 800CW secondary antibody (#926-32211) were purchased from Li-COR (USA). The secondary antibody was diluted at 1: 10,000 dilution using its provided diluent solution. 4’, 6’-diamidino-2-phenylindoledihydrochloride (DAPI) solution was purchased from Sigma-Aldrich (USA). CellTiter 96 Aqueous One Solution, an MTS kit, was purchased from Promega (USA).

*Cell culture*

Human colorectal adenocarcinoma cell line (SW480) and human normal colon cell line (CCD841) were purchased from American Type Culture Collection (ATCC, USA). The cells were cultured in DMEM medium (Gibco, USA) containing 10% fetal bovine serum (Gibco, USA) and 1% penicillin-streptomycin (Gibco, USA) at 37^o^C, 5% CO_2_ atmosphere. After reaching 80% confluence, the cells were sub-cultured. 

*Studying the binding ability of AS1411 aptamer by flow cytometry*

SW480 cells were seeded into 12-well plate at a density of 3×10^5 ^cells and further incubated for 24 hours at 37^o^C, 5% CO_2_ atmosphere. Then, the cells were incubated with 5 µM FAM-labeled ASA and FAM-labeled NBO. After 1 hour, the cells were washed four times with PBS and characterized by a BD FACSverse™ flow cytometer (BD Biosciences, USA). The percentage of cell that was labeled by the fluorescent dye was quantified.

*Studying the binding ability of AS1411 aptamer by fluorescence microscope*

Approximately 3×10^4^ cells of SW480 and CCD841 cells were seeded into each well of 96-well plates and further incubated for 24 hours at 37^o^C, 5% CO_2_ atmosphere. Next, the cells were treated with 10 µM FAM-labeled ASA and FAM-labeled NBO for 1 hour. Then, cell nuclei were stained with 1 mg/mL of DAPI solution (Sigma-Aldrich, USA) for 10 minutes and the cells were washed 4 times. To investigate the aptamer binding, the cells were observed and imaged by ECLIPSE Ts2R inverted fluorescence microscope (Nikon, USA). 

*RT-PCR of nucleolin and β-actin*

Total RNA was purified from cells using miRNeasy Micro Kit (Qiagen, USA), and reversely transcribed to cDNA using a High Capacity cDNA Reverse Transcription Kits (Applied Biosystems, USA). The mRNA levels of nucleolin and *β*-actin (internal control) were evaluated by RT-PCR. The primer sequences are shown in Supplementary Table 1.

*Preparation of Dox-loaded AS1411 aptamer*

ASA with the concentration of 5 µM was mixed with 0.95 µM Dox in phosphate-buffered saline (PBS) for 1.5 hour at room temperature to allow the intercalation of Dox at CG base pairing sites. Fluorescence signals from both free Dox and the intercalated Dox were measured by a Varioskan LUX microplate reader (Thermo Scientific, USA) (λ_Ex_ = 480 nm, λ_Em_ = 500-800 nm).

*Effect of AS1411 aptamer on the viability of SW480 cells*

SW480 cells were seeded approximately 5×10^3^ cells into each well of 96-well plates. After 24 hours, the cells were treated with ASA at designated concentrations for 6 days. The aptamer concentrations were 0, 3.125, 6.25, 12.5, 25, 50, 100, and 200 µM. Cell viability was determined by MTS assay according to the supplier’s protocol. The absorbance of the solution was determined at 490 nm using a microplate reader (Bio Tek, USA), and IC_50_ was calculated using GraphPad Prism 5 (GraphPad, USA).

*Toxicity of Dox on SW480 cells*

SW480 cells were seeded approximately 5×103 cells into each well of 96-well plates, and further incubated for 24 hours. Then, the cells were cultured in media containing Dox at the designated concentrations: 0, 0.5, 1, 1.5, 2, 2.5, and 3 µM. The cells were incubated with Dox for 48 hours before adding MTS solution. The absorbance of the solution was determined by the microplate reader and IC_50_ was calculated using GraphPad Prism 5.

*Effect of Dox-loaded AS1411 aptamer on SW480 and CCD841 cell growth*

SW480 and CCD841 cells were seeded approximately 5×10^3^ cells into each well of 96-well plates. After 24 hours, the cells were treated with following solutions; 5 µM ASA, 5 µM NBO, 0.95 µM Dox, 5 µM Dox-loaded NBO, and 5 µM Dox-loaded ASA. Molar ratios between Dox and ASA or NBO were 1 to 5. The treatment was carried out for 48 hours. Then cell viability was determined using MTS assay.

*Protein extraction and Western blot*

SW480 cells were treated with 5 µM NBO, 5 µM ASA, 0.95 µM Dox, 5 µM NBO loaded with 0.95 µM Dox, and 5 µM ASA loaded with 0.95 µM Dox. After cell collection, the pellets were washed three times with cold PBS, lysed with RIPA buffer (Ameresco, USA) containing protease inhibitors (Ameresco, USA), and sonicated until completely dissolved. Protein concentrations were measured using a Pierce BCA protein assay kit (Thermo Scientific, USA). The cell lysates were subjected to polyacrylamide gel electrophoresis (PAGE, resolving gel 12% and stacking gel 4%), and transferred to PVDF membrane by electrical blotting. After blocking with Odyssey blocking buffer for 1 hour at room temperature, the membranes were incubated with primary antibodies at 4^o^C overnight. After that, the membranes were incubated with secondary antibodies at room temperature for 1 hour. Band visualization and quantitation were performed using a LI-COR Odyssey Imager (Li-COR, USA).

*Statistical analysis*

All data were represented as mean ± standard deviation (SD) of at least triplicate samples. Comparisons between control and study groups were performed using Student’s t-test. Statistical significance was set at *P <0.05.

## Results

*Binding ability of AS1411 aptamer on SW480 cells*

**Figure 1:**
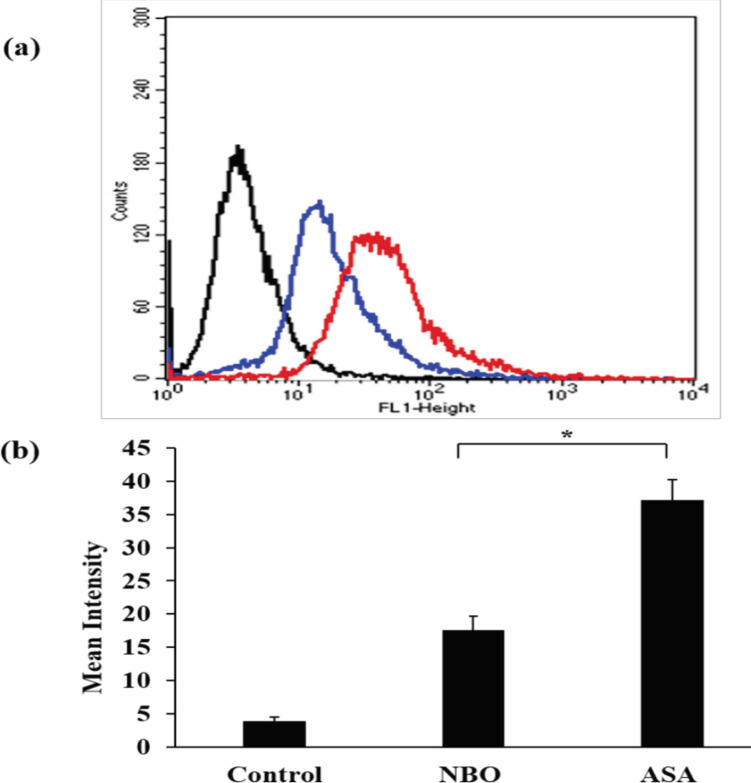
Specific Binding of ASA on SW480 Cells Examined by Flow Cytometry. (a) Histogram represents the number of SW480 cells treated with 5 µM FAM-labeled ASA (red line), 5 µM FAM-labeled NBO (blue line) and no treatment (black line). (b) Column chart indicates mean intensity ± SD in each treatment, * P < 0.05

To investigate the binding ability of ASA, SW480 cells treated with the aptamer and non-binding sequence were characterized by flow cytometry. From the histogram (Figure 1a), the vertical axis represents the number of analyzed SW480 cells, and the horizontal axis represents fluorescence intensity from FAM. Untreated SW480 exhibited relatively low fluorescence intensities from FAM. The cells treated with NBO showed slightly high fluorescence intensity from FAM due to non-specific binding, whereas the cells treated with ASA had higher fluorescence intensity from FAM than the other cell samples (Figure 1b). These results informed us that ASA was able to bind to SW480 cells, as demonstrated from fluorescence signal of FAM. The binding was facilitated by interaction between ASA and nucleolin (Reyes-Reyes et al., 2010; Mosafer and Mokhtarzadeh, 2018).

**Figure 2:**
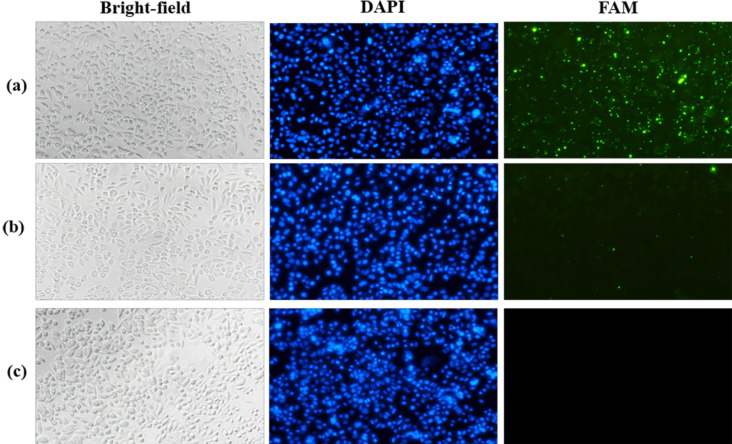
Specific Binding of ASA on SW480 Cells Observed under a Fluorescence Microscope at 20x Magnification. SW480 cells were treated with 10 µM FAM-labeled ASA (a), 10 µM FAM-labeled NBO (b), and without treatment (c). From the left to right column, representative images demonstrated a bright field, DAPI nuclear staining (blue), and FAM-labeled aptamers inside the cells (green).

**Figure 3:**
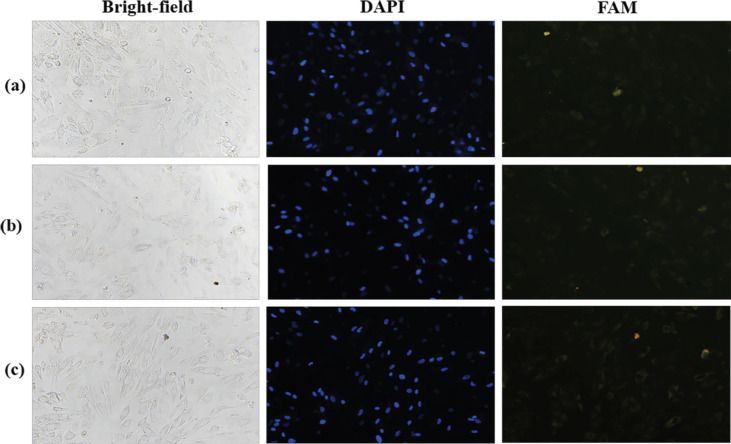
No Binding of ASA on CCD841 Cells Observed under a Fluorescence Microscope at 20x Magnification. CCD841 cells were treated with 10 µM FAM-labeled ASA (a), 10 µM FAM-labeled NBO (b), and without treatment (c). From the left to right column, representative images demonstrated a bright field, DAPI nuclear staining (blue), and FAM-labeled aptamers (green).

**Figure 4:**
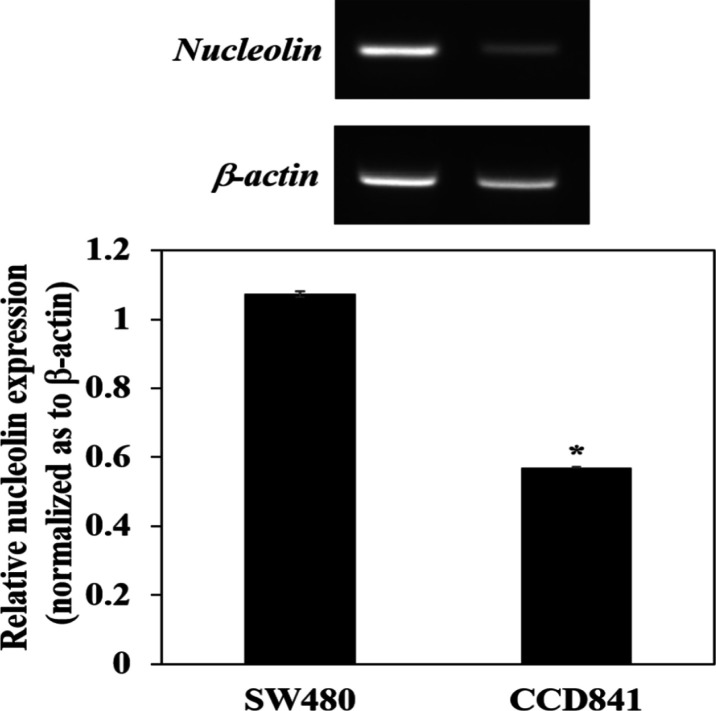
Nucleolin mRNA Expression in SW480 and CCD 841 CoN Cell Lines. The mRNA levels of nucleolin and β-actin were analyzed by Reverse Transcription-Polymerase Chain Reaction (RT-PCR) analysis. Bar graphs represent quantitative analysis of band intensities normalized as to β-actin. The values are presented as mean ± SD, *P < 0.05

To confirm binding capability of ASA, the aptamer was labeled with FAM, a fluorophore, and then it was used to treat SW480 cells. The treated cells were observed under fluorescence microscope. The result showed that SW480 treated with the aptamer demonstrated higher fluorescence signal than the cells treated with NBO (Figure 2). The binding specificity of ASA was further investigated using CCD841 cells for a comparison. The result showed that CCD841 cells treated with the aptamer indicated lack of fluorescence signal (Figure 3). The expression of nucleolin mRNA was significantly lower in CCD841 than that in SW480 cells (Figure 4). These results indicated that ASA bound specifically to SW480 cells via the interaction between this aptamer and the nucleolin highly expressed on the cell surface (Soundararajan et al., 2009). As mentioned in the literature, the binding capability of this aptamer was dependent on aptamer concentration, backbone chemistry, and cell type (Bates et al., 2009), and binding mechanism relied on receptor-mediated endocytosis (Juliano et al., 2008). 

*Effect of AS1411 aptamer on the viability of SW480 cells*

**Figure 5:**
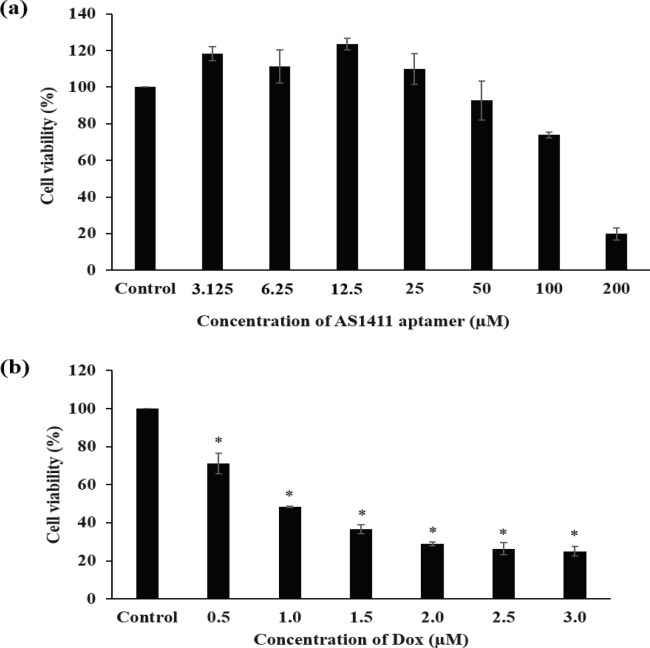
Effects of ASA and Dox on the Cell Viability of SW480. SW480 Cells were Treated with ASA (3.125, 6.25, 12.5, 25, 50, 100 and 200 µM) for 6 days (a), and Treated with Dox (0.5, 1, 1.5, 2, 2.5, and 3 µM) for 48 hours (b). The cell viability was measured using MTS assay. The values are presented as mean ± SD, n = 3, *P < 0.05 compared with the control

To assess the anti-proliferative effect of ASA, SW480 cells were treated with the aptamer at several concentrations for 6 days using methods reported in a publication (Ireson and Kelland, 2006). MTS assay was carried out to determine cell viability of the treated samples. The results showed that the ASA had no effect on the proliferation of SW480 cells when the aptamer concentrations were not greater than 12.5 µM. When concentrations of the aptamer were over 25 µM, the ASA could suppress cell growth in a concentration-dependent manner (Figure 5a). The half maximal inhibitory concentration (IC_50_) of ASA calculated by GraphPad Prism 5 was 144 µM. 

*Toxicity of Dox on SW480 cells*

Toxicity of Dox on SW480 cells was investigated by treating the cells with Dox at designated concentrations for 48 hours. Then MTS assay was used to measure the cell viability. The result showed that Dox was toxic to SW480 in a dose-dependent manner (Figure 5b). The IC_50_ value of Dox on SW480 cells was 0.95 µM, and it was further applied in following experiments about the intercalation of Dox into ASA. Dox, a chemotherapeutic drug for cancer treatment, is also toxic to normal cells resulting in a number of side effects to the patients (Carvalho et al., 2009). To overcome this shortcoming of Dox, strategies which relied on incorporating Dox into other molecules have been studied (Green and Rose, 2006; Liao et al., 2015; Zhao et al., 2018; Zhang et al., 2020a). In this research, we proposed a strategy for reducing Dox toxicity by intercalating the Dox into ASA (Dox-loaded AS1411 aptamer) and validated its functionality.

*Preparation of Dox-loaded AS1411 aptamer*

**Figure 6:**
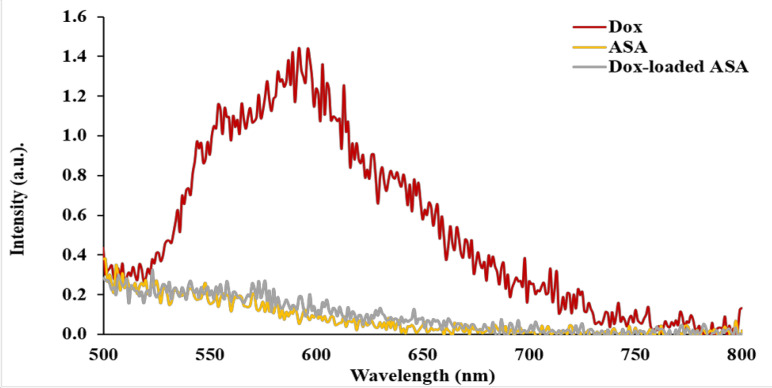
Intercalation of Dox into ASA Molecule. Dox with the concentration of 0.95 µM was incubated with 5 µM ASA in phosphate-buffered saline (PBS) for 1.5 hour at room temperature. A line graph represents fluorescence spectra of free Dox (red), ASA (yellow), and Dox-loaded ASA (gray).

Dox intercalated into ASA molecules was evaluated by fluorescence spectroscopy. The spectra of Dox, ASA, Dox-loaded ASA are shown in Figure 6. Dox emitted a maximum fluorescence signal at approximately 590 nm as reported in other research (Airoldi et al., 2014). Its fluorescence signal was lowered upon mixing with ASA, indicating the intercalation of Dox in base pairing sites of ASA. Molar ratio of Dox to ASA was 1:5 for a complete quenching and intercalation.

*Effect of Dox-loaded ASA on SW480 and CCD841 cell growth*

**Figure 7:**
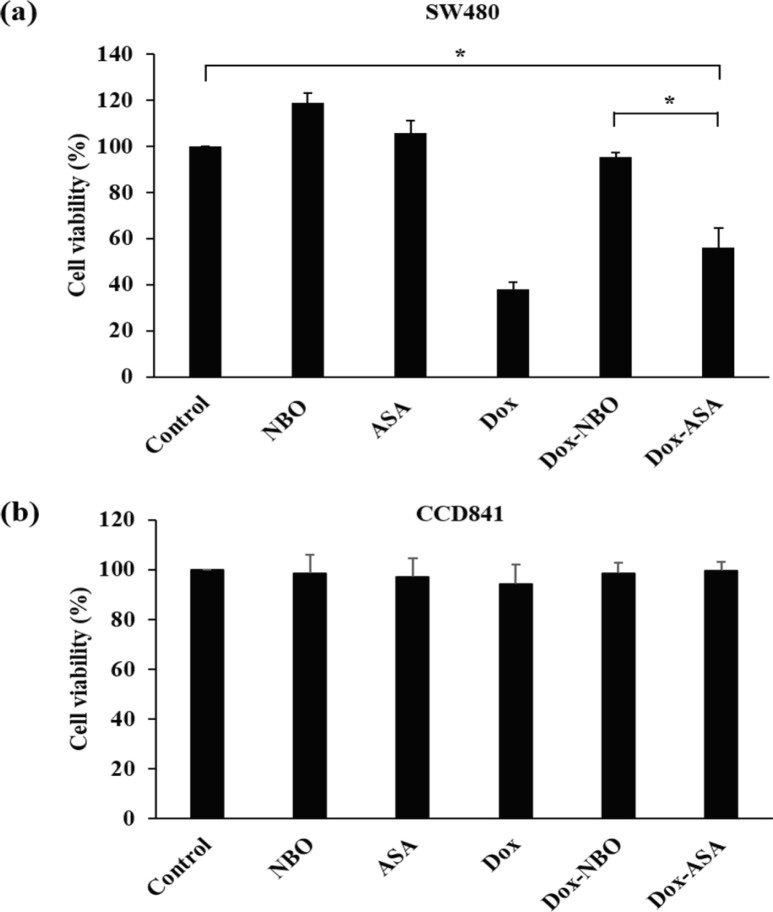
Effect of Dox-Loaded ASA on SW480 (a) and CCD841 (b) Cell Growth. Cells were treated with 5 µM ASA, 5 µM NBO, 0.95 µM Dox, 5 µM Dox-loaded NBO, and 5 µM Dox-loaded ASA for 48 hours. The cell viability was measured using MTS assay. The values are presented as mean ± SD, n = 3, *P < 0.05 compared with the control

Effect of Dox-loaded ASA on SW480 and CCD841 cell proliferation was evaluated by treating the cells by the following reagents: NBO, ASA, free Dox, Dox-loaded NBO, Dox-loaded ASA. Then MTS assay was used to measure cell viability. The SW480 cells treated with NBO and ASA had the level of cell viability comparable to the cells with no treatment, although the ASA alone did not obviously suppress cell viability in previous experiments (Figure 7a). 

When SW480 cells were cultured in the presence of free Dox, the cell viability had the lowest level compared to other tested samples because Dox was toxic to these cells. Dox-loaded NBO was non-toxic to SW480, since Dox was intercalated into oligonucleotide duplex having anionic nature which could prevent a cellular uptake of Dox (Tseng et al., 2009). Dox-loaded ASA exhibited toxicity to SW480 cells but it was less toxic than free Dox. The toxicity of Dox-loaded ASA came from the binding ability of AS1411 aptamer for bringing this toxic drug into the cells. Meanwhile the intercalation of Dox in the aptamer structure reduced the toxicity of Dox in cells as found in a previous study (Meng et al., 2012).

On the other hand, the cell proliferation assay showed that ASA, free Dox and Dox-loaded ASA had no effect on CCD841 cell growth, compared with the control group (Figure 7b). This indicated that ASA, free Dox, and Dox-loaded ASA were non-toxic to the normal colon cells. The result suggested that Dox-loaded ASA had the ability to selectively reduce CRC cell proliferation without affecting normal cells.

*Induction of apoptosis by Dox-loaded ASA via caspase-3*

**Figure 8:**
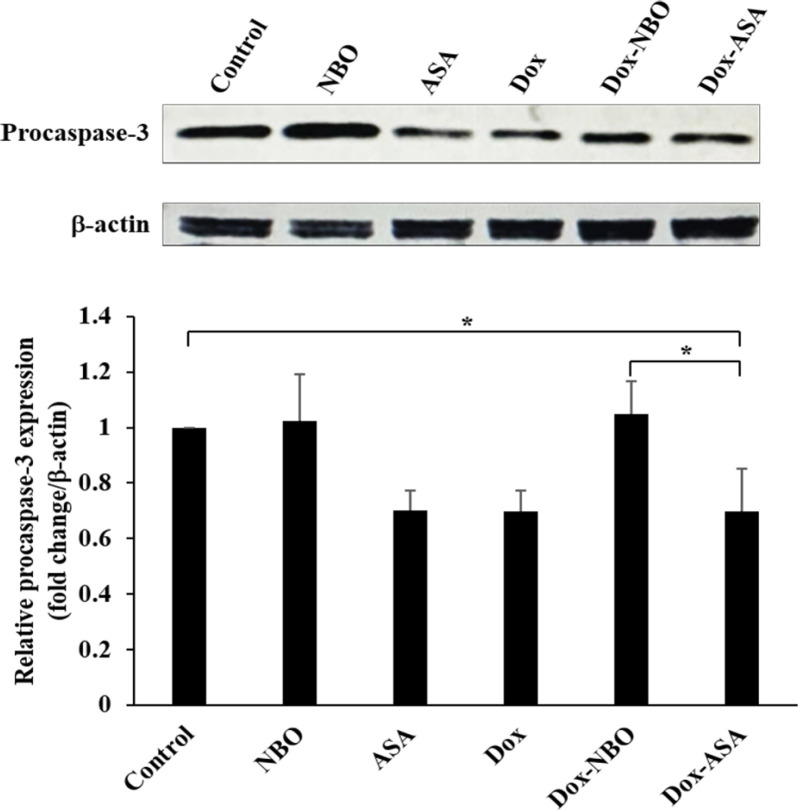
Decrease in Procaspase-3 Protein Level after Treatment with Dox-loaded ASA. SW480 cells were treated with NBO, ASA, Dox and combination, and the untreated cell served as control. The protein levels of procaspase-3 and β-actin were analyzed by western blot. Bar graphs represent quantitative analysis of band intensities normalized as to β-actin. The values are presented as mean ± SD, n = 3, *P < 0.05

To clarify whether Dox-loaded ASA induced cell apoptosis, the degradation of procaspase-3 was measured as an indicator since different upstream pathways leading to apoptosis depend on caspase-3 activation for final execution. Western blot analysis revealed that the level of procaspase-3 normalized with β-actin was significantly decreased after treatment of ASA, free Dox, and Dox-loaded ASA compared to the untreated cells (Figures 8). The result was consistent with the previous studies showing that inhibition of nucleolin with antisense oligodeoxynucleotides promoted apoptosis in nasopharyngeal cancer (Wu et al., 2012), and Dox could provoke cell apoptosis via caspase-3 activation in breast cancer (Pilco-Ferreto and Calaf, 2016). This finding suggested that Dox-loaded ASA induced apoptosis in SW480 cells mediated by the proteolytic cleavage of caspase-3. 

**Table 1:** Oligonucleotide Sequences. The Underline indicates AS1411 Sequence

Name	Sequence (5ˊ→3ˊ)
AS1411 aptamer (ASA)	GGTGGTGGTGGTTGTGGTGGTGGTGGCCATCGGCTATCGAAGCTCGAT
Non-binding oligonucleotide (NBO)	TTCCTCCTCCTCCTTCTCCTCCTCCTCCATCGGCTACTATCGAAGCTCGAT

## Discussion

Dox is an anti-cancer drug that is commonly used for treatment of many cancers. It damages the cells by poisoning topoisomerase-II, inhibiting DNA replication, and inducing the formation of reactive oxygen species in cells. This causes a number of side effects that make the treated patients suffer. A possible way to overcome this undesirable issue in using Dox is an integration of recognition elements to improve the ability of this drug to target specific corresponding sites. A recognition element of interest is an oligonucleotide, known as AS1411 aptamer that exhibits specific binding ability to nucleolin. This molecule is overexpressed in a number of cancer cells including SW480 cell, a human colorectal adenocarcinoma cell, which has been used as a representative cell in our CRC study.

Flow cytometer and fluorescence microscope were used to verify the binding of ASA to SW480 cells. Both assays gave a solid evidence that ASA bound to SW480 cells because the cells highly expressed nucleolin. RT-PCR results demonstrated that nucleolin was overexpressed in SW480 cells when compared to the CCD841 cells, confirming the findings of other studies showing the upregulation of nucleolin protein in several cancer cell lines (Dam et al., 2014). The expression of nuleolin protein in SW480 cells was reported in a previous study (Semba et al., 2010). This investigation suggested that nucleolin played an important role in the acceleration of CRC growth via a signaling of phosphatase of regenerating liver-3. In addition, the binding interaction between ASA and nucleolin enhanced the specificity of gold nanoparticles to CRC cells which was beneficial in chemophotothermal therapy (Zhang et al., 2020b). 

Cytotoxicity of ASA was another concern in applying this aptamer as chemotherapeutic agents. ASA exhibited anti-proliferative effect to a number of CRC such as HCC 2998, HT-29, KM12 cells (Bates et al., 2009). However, to the best of our knowledge, the growth inhibition of ASA to SW480 cells has not been reported. Cytotoxicity of ASA presented in this work might fill in the missing information. From acquired information about the cytotoxicity of ASA, the concentration of ASA used in preparation of Dox-loaded ASA was kept below the IC_50 _of ASA, so that the anti-proliferation of SW480 cells was caused by the drug formulation not the aptamer which was the main focus of this work. Moreover, cytotoxicity level of Dox reported in this work was in the same order of magnitude as the results from other research groups. For instance, after treating the cells with Dox for 24, 48, and 72 hours, the IC_50_ values were 65.25 µM (Xu et al., 2003), 0.35 µM (Zhang et al., 2020b) and 0.29 µM (Mielczarek-Puta et al., 2019), respectively. This might indicate that cytotoxicity of Dox increased with the longer treatment durations.

Fluorescence spectrometry was used to demonstrate the intercalation of Dox, because the fluorescence signal of Dox was quenched in DNA duplex. Dox intercalation proceeds through two moieties in the drug structure: an amino sugar group and a tetracyclic core. The amino sugar moiety interacts at AT sites of DNA duplex, while the tetracyclic is inserted in the GC sites (Perez-Arnaiz et al., 2014). The ASA sequence used in this work had a total of 48 nucleotides, of which the first 28 nucleotides starting from the 5’ end were in the nucleolin binding region, and the other 20 nucleotides were designated as sites for the intercalation of Dox.

Dox and ASA have been integrated in a number of materials such as aptamer-drug hybrids, liposomes, micelles, DNA nanostructures, polymers, and silver and gold nanoparticles. The anti-cancer functions of these systems have been tested toward liver, breast, cervical, prostate, and lung cancer (Yazdian-Robati et al., 2020). There have been few studies focusing on applying Dox and ASA to colorectal cancer. For instance, the DNA nano structure formed by four molecules of ASA was used to selectively deliver Dox into CT26 colon cancer cells and killed these cells effectively (Yao et al., 2020), but the effect of Dox and ASA formulation on cellular pathways of CRC required more studies to fill in the information. Our work demonstrated that Dox-loaded ASA inherited binding ability of the aptamer and cytotoxicity of the drug resulting in anti-proliferative effect toward CRC cells. No change was observed after exposure of normal colon cells to the same concentration as that used in CRC cells. The finding indicated that Dox-loaded ASA selectively killed cancer cells while sparing normal cells.

Apoptosis pathway investigated by western blot was proceeded through the determination of procaspase-3, a key apoptotic signaling molecule. The significant reduction of procaspase-3 was observed in SW480 cells treated with ASA, Dox, and Dox-loaded ASA, confirming that these drugs when used alone or in combination were able to activate caspase-3. The elevated expression of nucleolin, the specific target of ASA, was associated with several processes in carcinogenesis, including survival, proliferation, metastasis, and angiogenesis. Nucleolin promoted the anti-apoptosis by stabilizing Bcl-xL mRNA and preventing it from degradation in breast cancer (Wang et al., 2014). Besides, nucleoin was supposed to bind FAS, block the FAS/FASL interaction, and thus inhibit the FAS-mediated apoptosis in B-cell lymphoma (Wise et al., 2013). Since FAS signaling pathway was commonly impaired in chemoresistant cancers, using ASA combined with Dox might empower the treatment efficacy. Although Dox is widely used as an effective anti-cancer drug, its clinical use is limited by cardiotoxicity resulting in congestive heart failure. However, the evidence showed that Dox induced apoptosis in cancer and normal cells by different mechanisms. Dox caused apoptosis in cancer by activating p53 and then stimulating caspase-3, while hydrogen peroxide played a crucial role in apoptosis in endothelial and myocardial cells (Wang et al., 2004). To reduce the toxicity from Dox in normal cells, a combination of Dox-loaded ASA with some agents that can detoxify hydrogen peroxide such as redox-active metalloporphyrin and glutathione peroxidase is worth exploring further.

The development of targeted chemotherapeutic agents is an important task for improving the well-being of cancer related patients. As mentioned above, Dox, a traditional anticancer drug, has some vital side effects that limit its applications. Meanwhile, nucleic acid aptamers exhibiting recognition ability makes them the ideal molecules for enhancing the specificity of chemotherapeutic drugs. Through this work, the exploration of Dox-loaded ASA on CRC provides more understanding of our formulation in cellular levels, and the integration of Dox and ASA is a promising strategy that yields a safe and effective anti-cancer drug. 

## Author Contribution Statement

The authors confirm contribution to the paper as follows. Walaiporn Lohlamoh makes contributions on data acquisition, data analysis, and manuscript preparation. Boonchoy Soontornworajit makes contributions on research design and recommendation, data analysis, and manuscript preparation. Pichayanoot Rotkrua, a principal investigator and corresponding author, makes contributions on research design and recommendation, data acquisition, data analysis, and manuscript preparation. All authors reviewed the results and approved the final version of the manuscript.

## Data Availability

All data are available from the corresponding author on reasonable request.
